# Towards AI-driven prediction of *HTT* CAG size in super-expanded human spiny projection neurons from Huntington disease donors

**DOI:** 10.1177/18796397261443137

**Published:** 2026-04-20

**Authors:** Simone Maestri, Davide Scalzo, Martina Zobel, Dario Besusso, Elena Cattaneo

**Affiliations:** 1 Dipartimento di Bioscienze, 9304 Università degli Studi di Milano, 20133, Milano, Italy; 2 70301 Istituto Nazionale di Genetica Molecolare, Milan, Italy

**Keywords:** somatic instability, mathematical modelling, cell-autonomous transcriptional dysregulation, spiny projection neurons, single-nucleus transcriptome and matched HTT CAG size

## Abstract

Somatic instability (SI) of the CAG tract in *HTT* is a major driver of neurodegeneration of Spiny Projection Neurons (SPNs), the primary neuronal subtype affected in Huntington's disease (HD). SPNs can accumulate hundreds of CAG repeats during a patient's lifetime, and once the expansion exceeds ∼150 CAGs, they acquire distinct, cell-autonomous transcriptional alterations that ultimately contribute to degeneration. Here, we developed the “HD-Phase-Model”, a mathematical framework designed to identify “super-expanded” SPNs without repeat sizing, by leveraging the only available single-nucleus HD post-mortem dataset that provides both transcriptional profile and matched *HTT* CAG sizes. After validating model performance on the test data, we applied it to independent single-nucleus datasets lacking CAG sizing information and across multiple brain regions. In all cases, the model consistently detected SPNs populations with convergent transcriptional dysregulation signatures indicative of extreme CAG expansion.

Importantly, although the model was trained on caudate SPNs, we observed highly similar dysregulation patterns in putamen and accumbens, while no evidence of super-expansion was found in SPNs from Alzheimer's and Parkinson's disease donors.

Together, these findings demonstrate that transcriptional profiles alone can serve as predictors of *HTT* CAG size, enabling systematic identification of super-expanded SPNs and providing insights into HD-specific neurodegenerative mechanisms.

## Plain language summary abstract

In Huntington's disease (HD), certain brain cells called spiny projection neurons (SPNs) are especially vulnerable. One reason is that the gene involved in HD (called *HTT*) can gain extra DNA repeats, known as CAG repeats, over a person's lifetime. When these repeats grow very large—over about 150—they cause major changes in how SPNs work, eventually leading to cell damage and death.

To study this, we created a mathematical tool called the HD-Phase-Model. This model can identify SPNs with very large CAG repeat expansions just by looking at their gene activity patterns, without needing to directly measure repeat length. After confirming that the model worked correctly on known data, we applied it to several independent datasets from different brain regions. The model consistently detected SPNs showing the same harmful gene activity patterns linked to very large CAG expansions. Importantly, these patterns were specific to HD and were not found in brain cells from people with Alzheimer's or Parkinson's disease.

Overall, our work shows that patterns of gene activity can be used to predict which SPNs have extreme CAG expansions. This provides a new way to study how these expansions drive HD-specific brain cell damage.

## Graphical abstract

**Graphical Abstract:**
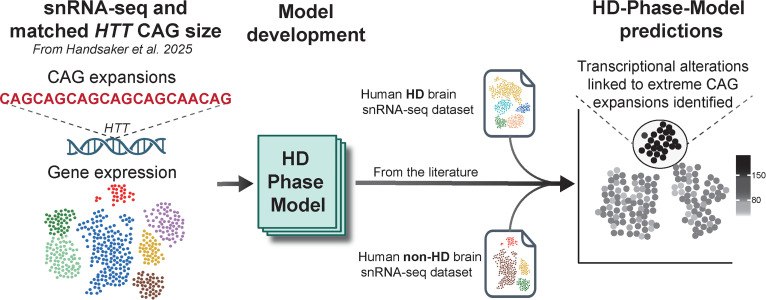
This is a visual representation of the abstract.

## Introduction

The unstable nature of the CAG tract in the exon 1 of *HTT*, the gene causing Huntington's disease (HD), was recognized at the time of its discovery.^1,2^ In the years that followed, it became clear that the CAG repeat undergoes expansion not only in the germline but also in somatic cells of the brain, a process known as somatic instability (SI).^3–6^ However, scientific interest in SI significantly increased only after Genome Wide Association Studies (GWAS) showed that some genetic variants can modulate HD age of onset (AOO) through mechanisms involving SI.^7–10^

Recent studies using post-mortem HD brains further advanced the field by showing that SI is not only tissue-specific,^11^ but also cell-type-specific.^12–14^ A key breakthrough came with the development of a novel library preparation method that enables simultaneous measurement of *HTT* CAG size and transcriptional profiles from matched single nuclei.^15^ Using this method, Handsaker and colleagues demonstrated that: (i) Spiny Projection Neurons (SPNs) in the striatum – the most vulnerable neuronal subtype and brain region in HD – exhibit the highest levels of SI, with some cells accumulating over 800 CAG repeats during a patient's lifetime; (ii) SPNs harbouring >150 CAGs (termed “super-expanded” SPNs) display additional, cell-autonomous transcriptional dysregulation compared to neighbouring, less-expanded SPNs from the same brain; (iii) super-expanded SPNs are marked by a set of genes referred to as “Phase C” and “Phase D” genes, which exhibit CAG-length-dependent transcriptional changes.^14^ Specifically, “Phase C” genes show expression levels that either linearly increase (Phase C+) or decrease (Phase C-) in SPNs with >150 CAGs, while “Phase D” genes – typically not expressed in SPNs – become aberrantly de-repressed when the number of CAG repeats largely surpasses the 150 CAGs threshold. Overall, these findings suggest that the presence of a fraction of SPNs with >150 CAGs is associated with – and may be sufficient to – trigger the HD neurodegeneration process. These findings were formalized into a conceptual framework called the ELongATE model (Extra Long repeats Acquire Toxic Effect), which describes the progressive, CAG-length-dependent transcriptional changes that occur in SPNs as somatic expansions increase. According to this model, the accumulation of super-expanded SPNs (in Phases C and D) reflects a critical molecular transition that may be sufficient to initiate HD neurodegeneration. Moreover, recent studies have provided evidence that reducing SI in SPNs below the 150 CAG threshold yields therapeutic benefits in HD mouse models and cell lines, thereby consolidating SI modulation as a potential target for future therapeutic strategies.^16–19^

In this work, we exploited the only available HD post-mortem dataset that includes matched single-nucleus transcriptional profiles and CAG sizing to build a mathematical model capable of predicting the *HTT* repeat size of individual SPNs. After validating accurate performance of the model on a subset of the same dataset, we applied it to external single-nuclei datasets covering multiple brain regions from postmortem HD and non-HD samples. We show that the model identifies a subset of SPNs in these external HD datasets that exhibit cell-autonomous transcriptional dysregulation signatures typical of super-expanded SPNs, and that these alterations are not limited to the caudate nucleus, but extend to SPNs from the accumbens and putamen as well. Notably, the model did not detect SPNs with super-expanded transcriptional characteristics in SPNs from Alzheimer's and Parkinson's disease donors. Thus, by leveraging transcriptional profiles alone, our model provides a powerful tool to infer *HTT* CAG repeat size at the single-cell level and to uncover the contribution of super-expanded SPNs to HD pathogenesis.

## Materials and methods

### Handsaker et al. dataset

Single-nuclear sequencing data were downloaded for Handsaker et al.,^14^ and Seurat objects with read counts for each sample were loaded into an R v4.3.0 environment using *Read_10X_h5*, *SingleCellExperiment* and *CreateSeuratObject* functions from Seurat v5.0.0 R package.^20^ Only cells annotated as “SPN” were retained with the *subset* function and Seurat objects from each sample were then merged with *merge* function. Counts were normalized, scaled and variable features were identified with *SCTransform* and then dimensionality reductions were obtained with *RunPCA* and *RunUMAP* functions from the Seurat package. UMAPs were plotted using *FeaturePlot* and *DimPlot* functions. As a quality control, individual cells in the UMAP were coloured by donor, number of expressed genes, number of detected molecules, mitochondrial genes percentage and number of CAG repeats in the *HTT* gene (Figure S1A-E). Using *FindNeighbors* and *FindClusters* functions with 0.01 resolution, two main clusters were identified in the UMAP (Figure S1F). The distribution of CAG sizes for the expanded allele of SPNs from HD donors was plotted with *geom_histogram* from ggplot2 v3.5.0 package.^21^ SPNs from all donors were iteratively split into training and test sets, by training the model on SPNs from all donors except one, and testing the model on SPNs from the held-out donor. SPNs in the training set with their expanded allele CAG sized (>36 CAG) were retained and used to train a cross-validated logistic regression “Phase model” with *cv.glmnet* function from glmnet v4.1-8 package^22^ (family = “binomial”), to predict the probability of each SPN being in Phase ‘C-D-E’, based on the expression value of Phase C and Phase D genes, obtained from Handsaker et al..^14^ For each held-out donor, the model corresponding to the value of λ that gave minimum mean cross-validated error (“lambda.min”) was chosen and the classification probability threshold providing at least 99% precision on the training set was identified and used for classification on both training and test sets. Models performances were assessed on both training and test sets by plotting Precision-Recall (PR) curves and their PR-AUC, together with precision, recall and F1 score at the chosen probability classification threshold. The final “Phase model” to be later used on external datasets was then trained by exploiting SPNs from all donors and the classification threshold was set to 0.86 to reach 99% precision on the training set. The probability of each SPN being in Phase ‘C-D-E’ and the CAG size of each SPN were evaluated with *predict* function from glmnet package. For the “CAG sizing model”, SPNs with >150 CAG from all donors were iteratively split into training and test sets using a held-out-donor approach, and used to train a cross-validated linear regression model (family = “gaussian”) with *cv.glmnet* function from glmnet package.. Again, the model with “lambda.min” was selected. Models performances were assessed on both training and test sets by computing the Spearman correlation between measured and predicted CAG sizes, as well as residuals-based metrics, including RMSE, MAE, bias and R^2^. The final “CAG sizing” model was then trained on SPNs with >150 CAG from all donors. Weighted Pearson correlation values were evaluated between pairs of features with *wtd.cor* function from weights v1.1.2 R package,^23^ with the number of SPNs from each donor used as weight. Scatter plots were obtained with *geom_point* from ggplot2 or *smoothScatter* from graphics^24^ v3.6.2 R packages.

### Paryani et al. dataset

Single-nuclear sequencing data were downloaded from Paryani et al.^25^ and imported into an R environment with *readRDS, SingleCellExperiment* and *CreateSeuratObject* functions. Only cells labelled as “iSPN_1”, “iSPN_2”, “dSPN_1” and “dSPN_2” were retained with *subset* function. Moreover, SPNs from the available brain regions (caudate and accumbens) were split and analysed separately. For each condition and tissue, counts from each SPN were normalized, scaled and variable features were identified using *SCTransform* function, regressing out “nCount_RNA” and “nFeature_RNA” variables. *RunPCA* and *RunUMAP* functions were then used to obtain dimensional reductions. Given the lack of good batch integration in the UMAP, *RunHarmony* with group.by.vars = “Batch” and RunUMAP functions were used. UMAPs were plotted using *FeaturePlot* and *DimPlot* functions. The “Phase model” was run on SPNs from the two conditions and tissues, using the previously set classification threshold of 0.86.

### Lee et al. dataset

Single-nuclear sequencing data were downloaded from Lee et al.^26^ and imported into an R environment with *Read10X, SingleCellExperiment* and *CreateSeuratObject* functions*.* Only cells labelled as “D1_MSN” or “D2_MSN” were retained with *subset* function. Moreover, SPNs from the available brain regions (caudate and putamen) were split and analysed separately. For each condition and tissue, counts from each SPN were normalized, scaled and the variable features were identified using *SCTransform* function, regressing out “nCount_RNA”, “nFeature_RNA” and “percent.mt” variables. *RunPCA* and *RunUMAP* functions were then used to obtain dimensional reductions. Given the lack of good batch integration in the UMAP, *RunHarmony* with group.by.vars = “Batch” and RunUMAP functions were used. UMAPs were plotted using *FeaturePlot* and *DimPlot* functions. The “Phase model” was run on SPNs from the two conditions and tissues, using the previously set classification threshold of 0.86.

### Xu et al. dataset

Single-nuclear sequencing data were downloaded from Xu et al.^27^ and imported into an R environment with *Read10X*, *SingleCellExperiment* and *CreateSeuratObject* functions*.* Cells with a number of counts comprised between 5000 and 30000, a number of expressed genes comprised between 500 and 8000 and a percentage of mitochondrial transcripts below 10% were retained. Counts from each nucleus were normalized, scaled and the variable features were identified using *SCTransform* function, regressing out “nCount_RNA”, “nFeature_RNA” and “percent.mt” variables. *RunPCA* and *RunUMAP* functions were then used to obtain dimensional reductions. Given the lack of good batch integration in the UMAP, *RunHarmony* with group.by.vars = “SAMPLE” and *RunUMAP* functions were used. By exploiting functions *FeaturePlot*, *FindNeighbors* and *FindClusters* with resolution 0.1, clusters with cells expressing either DRD1 or DRD2 – corresponding to clusters “0” and “4” – were identified as SPNs and were retained. SPNs were split by condition (CTRL, AD and PD) and UMAPs were plotted using *FeaturePlot* and *DimPlot* functions, to confirm good samples integration. The “Phase model” was run on SPNs from the three conditions, using the previously set classification threshold of 0.86.

## Results

### Independent validation of transcriptional signatures of super-expanded SPNs in the caudate

**Figure 1.**
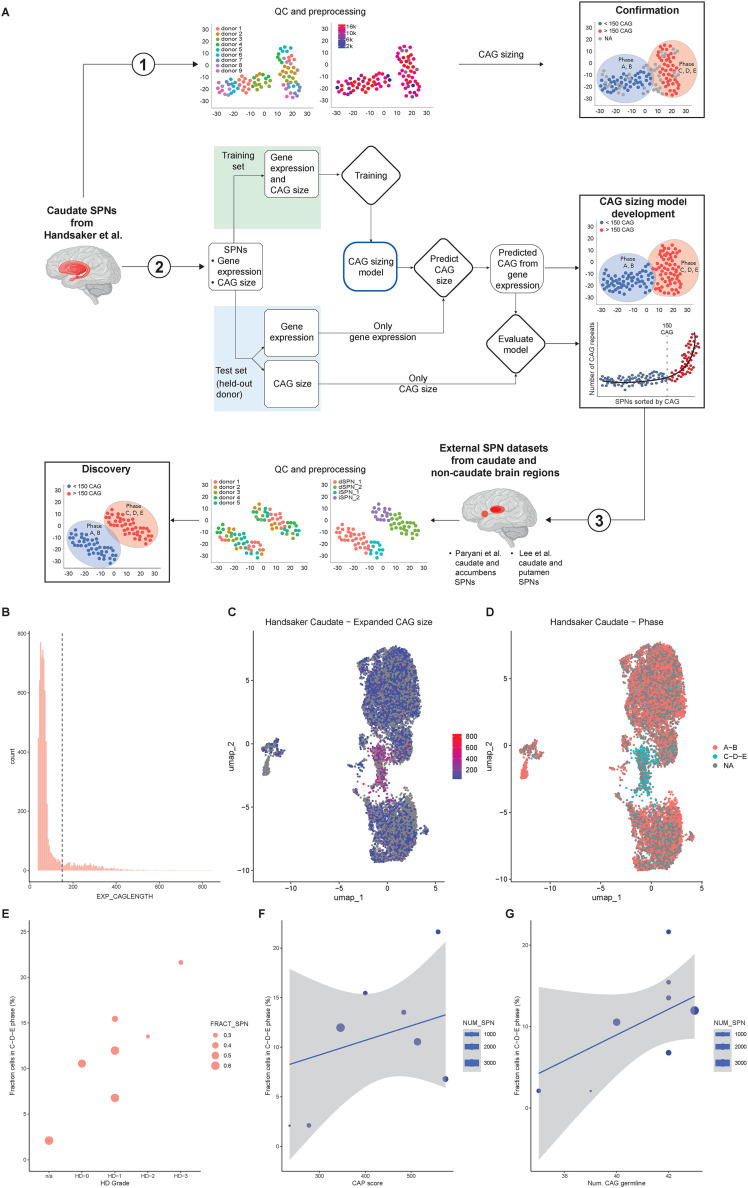
Extreme CAG expansions in caudate SPNs induce cell-autonomous transcriptional alterations. (A) Experimental study design; Caudate SPNs from Handsaker et al. from all donors are collectively analysed to confirm that, although CAG sizing of the expanded allele is not available for all SPNs (see SPNs depicted in grey), super-expanded SPNs co-localize on a UMAP; transcriptional profile and CAG size are iteratively split into training and test set to set-up and validate the HD-Phase-Model; the model is then applied to external datasets from multiple brain regions to discover SPNs predicted to carry extreme CAG expansions based on their transcriptional profile. (B) CAG size distribution of the expanded alleles of SPNs (>36 CAG); the dotted line marks the 150 CAG threshold. (C) UMAP of SPNs coloured by CAG size (legend); SPNs with < 36 CAG are set to ‘NA’ and coloured in grey. (D) UMAP of SPNs coloured by phase, after binarization of CAG size; SPNs with number of CAG between 36 and 150 are in ‘A-B’ phase; SPNs with > 150 CAG are in ‘C-D-E’ phase. SPNs with < 36 CAG are set to ‘NA’. (E) Fraction of SPNs in ‘C-D-E’ phase for each donor, grouped by HD grade. The size of each dot is proportional to the fraction of SPNs over all cell types. (F) Correlation between CAP score and fraction of SPNs in ‘C-D-E’ phase for each donor (r = 0.32, p-value = 0.43, 95% CI: [-0.49; 0.83]). (G) Correlation between number of CAG repeats in the germline for each donor and fraction of cells in ‘C-D-E’ phase (r = 0.58, p-value = 0.13, 95% CI: [-0.21; 0.91]).

Handsaker et al.^14^ recently showed that expansions beyond 150 CAG repeats are required to trigger cell-autonomous transcriptional changes in remaining SPNs of the same donor. Specifically, they grouped SPNs from each donor based on their CAG size to obtain an allelic series with identical genetic background and observed the same pattern across donors. As an independent validation, we first sought to confirm their results by jointly analyzing the transcriptional profiles and matched CAG lengths of SPNs from all donors collectively, rather than performing separate analyses for each donor (Figure 1**A**, branch #1). This approach was designed to assess whether the transcriptional alterations associated with extreme CAG expansions are primarily cell-autonomous and stronger than the difference due to donor-specific genetic backgrounds. Specifically, we wanted to determine whether these alterations drive the co-regionalization of SPNs on a UMAP, resulting in a homogeneous distribution of cells from each HD donor. We also investigated whether the proportion of SPNs carrying more than 150 CAG repeats correlates with clinically relevant parameters, such as the Vonsattel grade and the CAG-Age Product (CAP) score. The Vonsattel grade is a neuropathological classification system used to assess the severity of striatal degeneration in post-mortem HD brains, based on both macroscopic and microscopic criteria, and ranges from 0 to 4, in ascending order of severity.^28^ In contrast, the CAP score is a clinical index that combines the patient's age at study entry with the number of inherited CAG repeats, and is used to estimate the time to HD symptom onset.^29^

Single-nuclei RNA sequencing data for SPNs from 8 post-mortem HD brains and 1 non-HD brain, measuring both the *HTT* CAG repeat length and transcriptional profiles within the same nuclei, were downloaded from Handsaker et al..^14^ After quality control (Figure S1A-F), we assessed the distribution of CAG repeat lengths in the expanded *HTT* allele (Figure 1B). On average, 10.3% of SPNs per sample from HD donors carried more than 150 CAG repeats; this fraction increased to 13.2% when excluding the two donors sampled before the onset of HD symptoms. We then visualized the transcriptional profiles of SPNs using UMAP, with cells coloured according to the number of CAG repeats in the expanded *HTT* allele (Figure 1C). Notably, a distinct UMAP region was enriched for SPNs with >150 CAG repeats, suggesting that extreme CAG expansions are associated with cell-autonomous transcriptional dysregulation, compared to other SPNs from the same HD brains, shared by all donors.

To further investigate this, we assigned each SPN a ‘Phase’ of the ‘ELongATE’ model based on the CAG size of the expanded *HTT* allele, i.e., Phase ‘C-D-E’ for SPNs with >150 CAG repeats, Phase ‘A-B’ for SPNs with CAG repeats between 36 and 150 and ‘NA’ for SPNs with <36 CAG repeats, that had only the wild-type allele CAG sized. The impact of >150 CAG repeats on transcriptional identity became even more pronounced in this framework (Figure 1D). The proportion of SPNs in Phases ‘C-D-E’ (i.e., super-expanded SPNs) ranged from 1.6% in a pre-manifest HD donor to 21.5% in a donor with HD-3 Vonsattel grade (Figure 1E). This fraction showed a moderate positive although not significant correlation with CAP score (r = 0.32, p-value = 0.43, 95% CI: [-0.49; 0.83]) and with the number of CAG repeats in the germline (r = 0.58, p-value = 0.13, 95% CI: [-0.21; 0.91] for HD donors only; r = 0.76, p-value = 0.02, 95% CI: [0.21; 0.95] if including also the non-HD donor) (Figure 1F,G). Given the wide confidence intervals and lack of statistical significance within HD donors, these observations should be considered exploratory and do not support causal or robust associations, although they may suggest trends that warrant further investigation.

Taken together, these results confirm that the number of CAG repeats in the germline and age – along with other factors that may include environment, genetic background, and expression levels of trans-modifiers – potentially contribute to the emergence of super-expanded SPNs, which in turn was recently proposed to be associated with clinical parameters.

### The “HD-phase-model”: Predicting extreme CAG expansions from phase C/D gene expression

After independently confirming that SPNs with more than 150 CAG repeats in *HTT* exhibit a distinct transcriptional profile compared to other SPNs from the same post-mortem HD brains,^14^ we investigated whether the expression levels of Phase C and Phase D genes could be used to predict the number of CAG repeats in individual SPNs (Figure 1A, branch #2).

**Figure 2.**
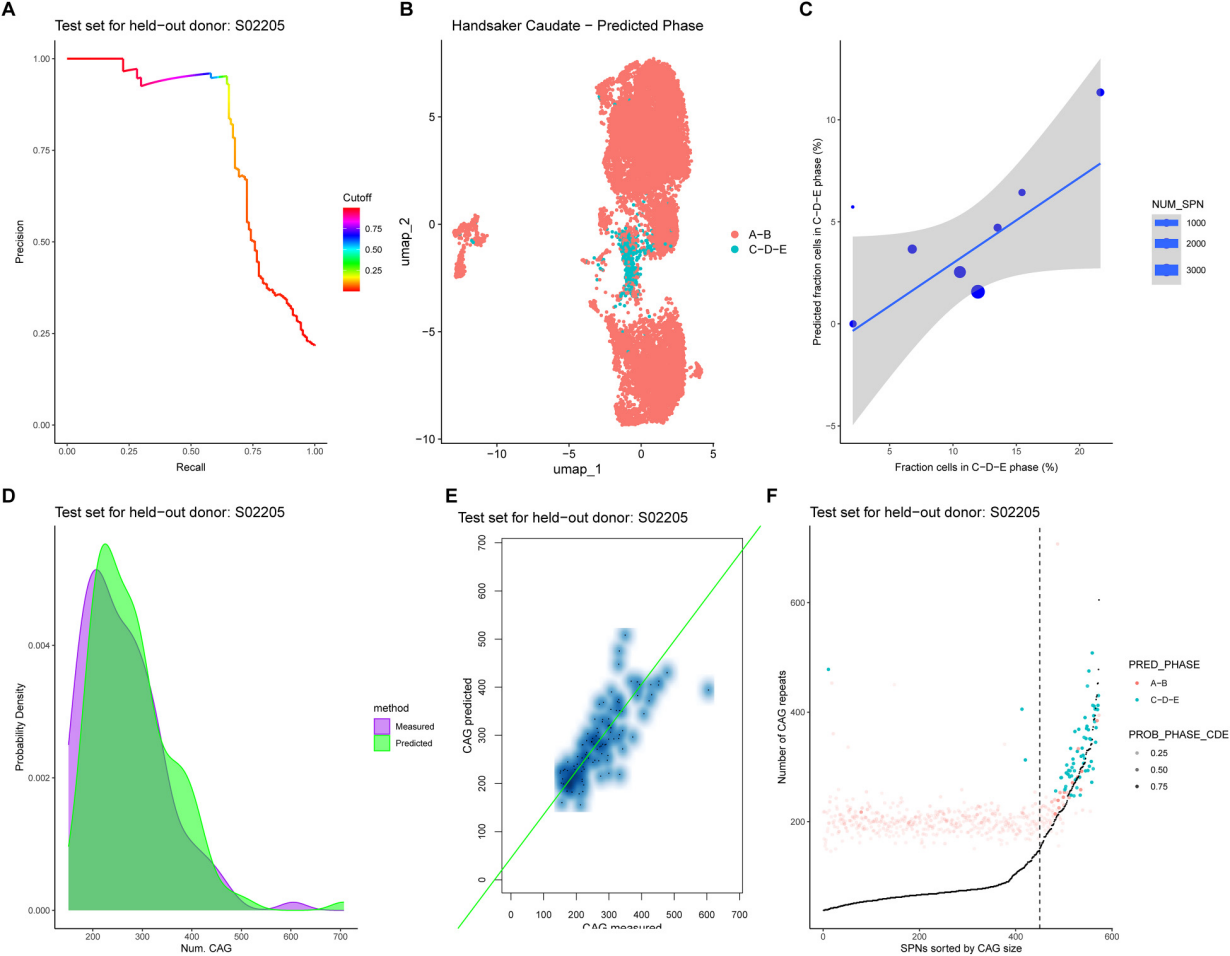
Predicting extreme CAG expansions in caudate SPNs based on the expression value of phase C and phase D genes. (A) PR curve on the test set for held-out donor S05202. (B) UMAP of SPNs coloured by predicted phase on SPNs from held-out donors, after binarization of CAG size; SPNs with number of CAG repeats between 36 and 150 are in ‘A-B’ phase; SPNs with > 150 CAG are in ‘C-D-E’ phase. (C) Correlation between measured and predicted fraction of SPNs for each donor, considering predictions from test sets of held-out donors, i.e., not used for training the models (r = 0.75, p-value = 0.02, 95% CI: [0.17; 0.94]). (D) Measured and predicted CAG size distribution of expanded alleles for SPNs in phase ‘C-D-E’ in test set of held-out donor S02205, i.e., not used for training the model. (E) Correlation between number of measured and predicted CAG repeats for SPNs in phase ‘C-D-E’ in test set of held-out donor S02205 (n = 72, r Spearman = 0.75, p-value = 0.00, 95% CI: [0.63; 0.84). (F) SPNs in the test set of held-out donor S02205 are sorted by measured CAG size and for each SPN the number of CAG repeats predicted by the model, coloured by predicted phase, is reported. Dots transparency is set depending on the predicted probability for each SPN of being in ‘C-D-E’ phase. Moreover, the measured number of CAG repeats is reported in black. A vertical dotted line is reported at the 150 CAG repeats threshold.

Given the limited availability of SPNs with sized expanded *HTT* allele (6611 SPNs in total, all from a single study^14^), and the substantial imbalance between SPNs in Phase ‘A-B’ vs those in Phase ‘C-D-E’ (only 747 out of 6611 SPNs had >150 CAG repeats), we employed a two-step modelling approach to build our “HD-Phase-Model” (Figure S2). After iteratively splitting the dataset into training and test sets by using a held-out-donor approach, we filtered each training set to include only SPNs with expanded alleles CAG sized and trained a logistic regression model (“Phase model”) to predict the probability of each SPN belonging to Phase ‘C-D-E’, based on the expression levels of Phase C and Phase D genes (Figure S2A). We repeated this procedure for all donors and set a probability classification threshold to achieve 99% precision on each training set. We then assessed the performance of each “Phase-Model” on both the training and test sets by reporting Precision-Recall curves (Figure 2A) together with performance metrics (Table S1, Table S3) and confusion matrices ( **Table S2** , Table S4) at the selected classification threshold. It is worth noting that, given the chosen high-precision classification threshold, the method is intended as a high-confidence detector of super-expanded-like transcriptional identity and may miss a subset of true positives.

We then retrieved “Phase-Model” predictions for all SPNs from iterative application of the model on held-out donors, including those SPNs with only the wild-type allele CAG sized, and coloured SPNs in the UMAP according to their predicted ‘ELongATE’ phase. SPNs predicted to be in Phase ‘C-D-E’ formed a distinct cluster on the UMAP, consistent with regions enriched in SPNs with measured CAG expansions >150 (Figure 2B). When considering only SPNs in the test sets with the expanded allele CAG sized, the model correctly predicted the ELongATE phase for 94% of SPNs. Moreover, the fraction of SPNs predicted to be in Phase ‘C-D-E’ strongly correlated with the measured fraction across donors, confirming the Phase-Model's accuracy on previously unseen data (r = 0.75, p-value = 0.02, 95% CI: [0.17; 0.94]) (Figure 2C).

We next trained a second model – a linear regression “CAG sizing model” – using only SPNs with >150 CAG repeats, to predict the number of CAG repeats based on Phase C and D gene expression levels (Figure S2B). Again, we iteratively held out one donor, trained the model on SPNs from the remaining donors, and evaluated its performance on both the training and test sets. After visually inspecting the distribution and the agreement between measured and predicted CAG sizes for super-expanded SPNs (Figure 2D-E), we systematically assessed model performance by computing the Spearman correlation between measured and predicted CAG sizes, along with selected residual-based metrics on both the training (Table S5) and test sets (Table S6).

Lastly, to visually represent the combined output of the two modules of the “HD-Phase-Model”, we applied them to SPNs from the test sets (Figure S2C) and visualized the combined predictions alongside the measured CAG sizes (Figure 2F). As expected, the majority of SPNs with <150 CAG repeats were accurately classified as ‘Phase A-B’ by the Phase model, and the CAG sizing model's predictions began to align with measured number of CAG repeats as they surpassed the 150 repeats threshold, providing approximate estimation of CAG length among detected super-expanded cells. In summary, the HD-Phase-Model infers extreme CAG expansions from Phase C/D gene expression (Figure 1A, branch #2).

### The HD-phase-model reveals HD-specific SPN sub-populations in external datasets

**Figure 3.**
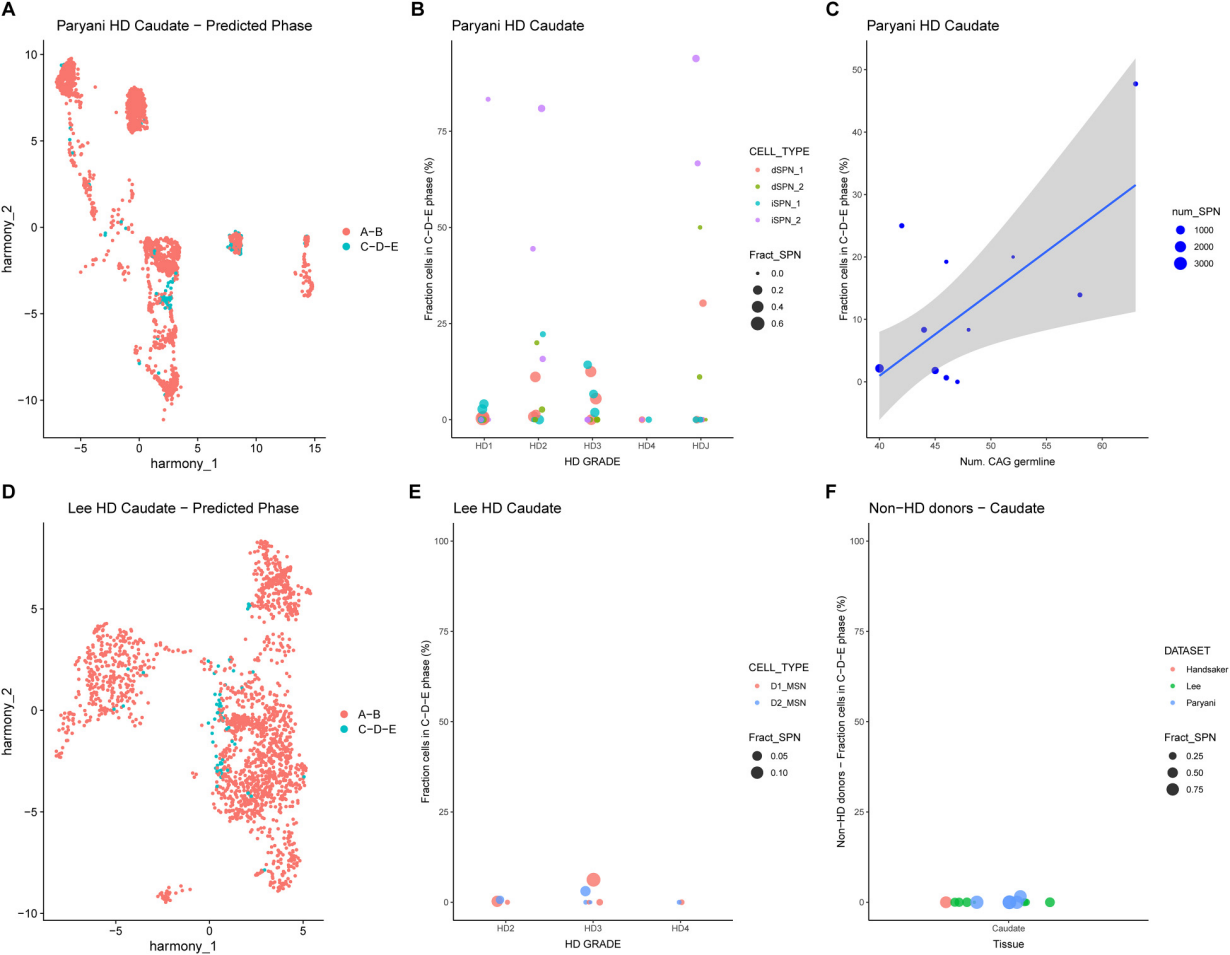
Caudate SPNs from external datasets predicted to be in ‘C-D-E’ phase share similar transcriptional profile. (A) UMAP of caudate SPNs from Paryani et al. coloured by predicted phase. (B) Dotplot showing the fraction of caudate SPNs from Paryani et al. in ‘C-D-E’ phase for each donor, with the size of each dot proportional to the fraction of SPNs over all cell types. Donors are grouped by HD Grade and are coloured by SPN subtype. (C) Correlation between number of CAG repeats in the germline of each donor and fraction of caudate SPNs from Paryani et al. in ‘C-D-E’ phase, with the size of each dot proportional to the number of SPNs (r = 0.70, p-value = 0.02, 95% CI: [0.61; 0.91]). (D) UMAP of caudate SPNs from Lee et al. coloured by predicted phase. (E) Dotplot showing the predicted fraction of caudate SPNs from Lee et al. in ‘C-D-E’ phase for each donor, with the size of each dot proportional to the fraction of SPNs over all cell types. Donors are grouped by HD Grade and are coloured by SPN subtype. (F) Dotplot showing the predicted fraction of caudate SPNs in ‘C-D-E’ phase for non-HD donors from Handsaker et al., Paryani et al. and Lee et al., with the size of each dot proportional to the fraction of SPNs over all cell types. Donors are coloured by dataset of origin.

After demonstrating accurate identification of super-expanded SPNs from Handsaker et al.,^14^ we applied our HD-Phase-Model to caudate SPNs from post-mortem HD donors in external datasets, generated using conventional single-nuclear short-read sequencing, i.e., lacking matched *HTT* CAG sizing (Figure 1A, branch #3). The studies by Paryani et al.^25^ and Lee et al.^26^ are traditional case-control studies, in which cells from multiple post-mortem brain areas of both HD and non-HD donors are sequenced at the single-nucleus level. This approach enables cell-type annotation and the identification of transcriptional alterations associated with HD at the cell-type level. Specifically, we subset caudate SPNs from 18 (13 HD, 5 CTRL) and 13 (6 HD, 7 CTRL) post-mortem donors reported in Paryani et al.^25^ and Lee et al.,^26^ respectively, and visualized their transcriptional profiles using UMAP. After verifying successful batch integration (Figure S3A,D), we examined the UMAP distributions based on finer cell-type annotations: dSPN_1, dSPN_2, iSPN_1 and iSPN_2 in the Paryani et al., and D1 and D2 SPNs in the Lee et al. dataset (Figure S3B,E). Interestingly, the iSPN_2 cluster identified by Paryani et al. was reported to be enriched in juvenile onset HD patients, suggesting that these iSPN exhibit transcriptional alterations associated with larger CAG repeat expansions. We also annotated cells by Vonsattel grade, ranging from HD1 to HD4 (Figure S3C,F). We next applied the HD-Phase-Model to SPNs from both datasets. We subset SPNs from HD donors and coloured them in the UMAP based on predicted ELongATE phase (Figure 3A,D). In both datasets, SPNs predicted to be in Phase ‘C-D-E’ formed distinct regions in UMAP space, consistent with the presence of cell-autonomous transcriptional dysregulation driven by extreme CAG expansions. To quantify this, we computed the fraction of Phase ‘C-D-E’ SPNs for each donor and sub-cell-type, grouping donors by Vonsattel grade. Interestingly, only iSPN_2 clusters from four donors showed more than 55% of cells predicted to be in Phase ‘C-D-E’. This suggests that the “_2” suffix in Paryani et al. may label subpopulations undergoing cell-autonomous transcriptional dysregulation due to extreme CAG expansions. It also explains the reduced representation of the iSPN_2 cluster in control donors - these are likely the subpopulations that emerge and expand specifically in the context of HD. However, both technical factors (such as sample preparation, library preparation methods and cell-type annotation) and biological factors (including age at death, CAG size in the germline, and genetic variants affecting SI) may influence the number of SPNs detected per donor – as shown by the size of each dot in Figure 3B,E – and, ultimately, the fraction of super-expanded SPNs. These factors may help explain the observed variability – even within the same HD grade.

Notably, in support of this hypothesis we found a strong positive correlation between the number of *HTT* CAG repeats in the germline of HD donors from Paryani et al. and the fraction of predicted Phase ‘C-D-E’ SPNs (r = 0.70, p-value = 0.02, 95% CI: [0.61; 0.91]), suggesting that higher germline CAG repeat numbers may accelerate HD onset, possibly via a SI-mediated neurodegenerative process (Figure 3C). Unfortunately, this information was not available for Lee et al.. As a negative control, we applied our HD-Phase-Model to caudate SPNs from 13 non-HD donors across the three datasets (Figure S4A-C) and found that 99.92% of SPNs were correctly predicted to be in Phase ‘A-B’, further validating the specificity and robustness of the Phase model. We confirmed the same observations from the Phase classification based on the threshold cut-off, by plotting UMAPs of caudate SPNs stratified by condition (HD vs CTRL), with each SPN coloured according to the inferred probability of being in Phase ‘C-D-E’ (Figure S5A-F).

### The HD-phase-model reveals transcriptional dysregulation in super-expanded SPNs across accumbens and putamen

**Figure 4.**
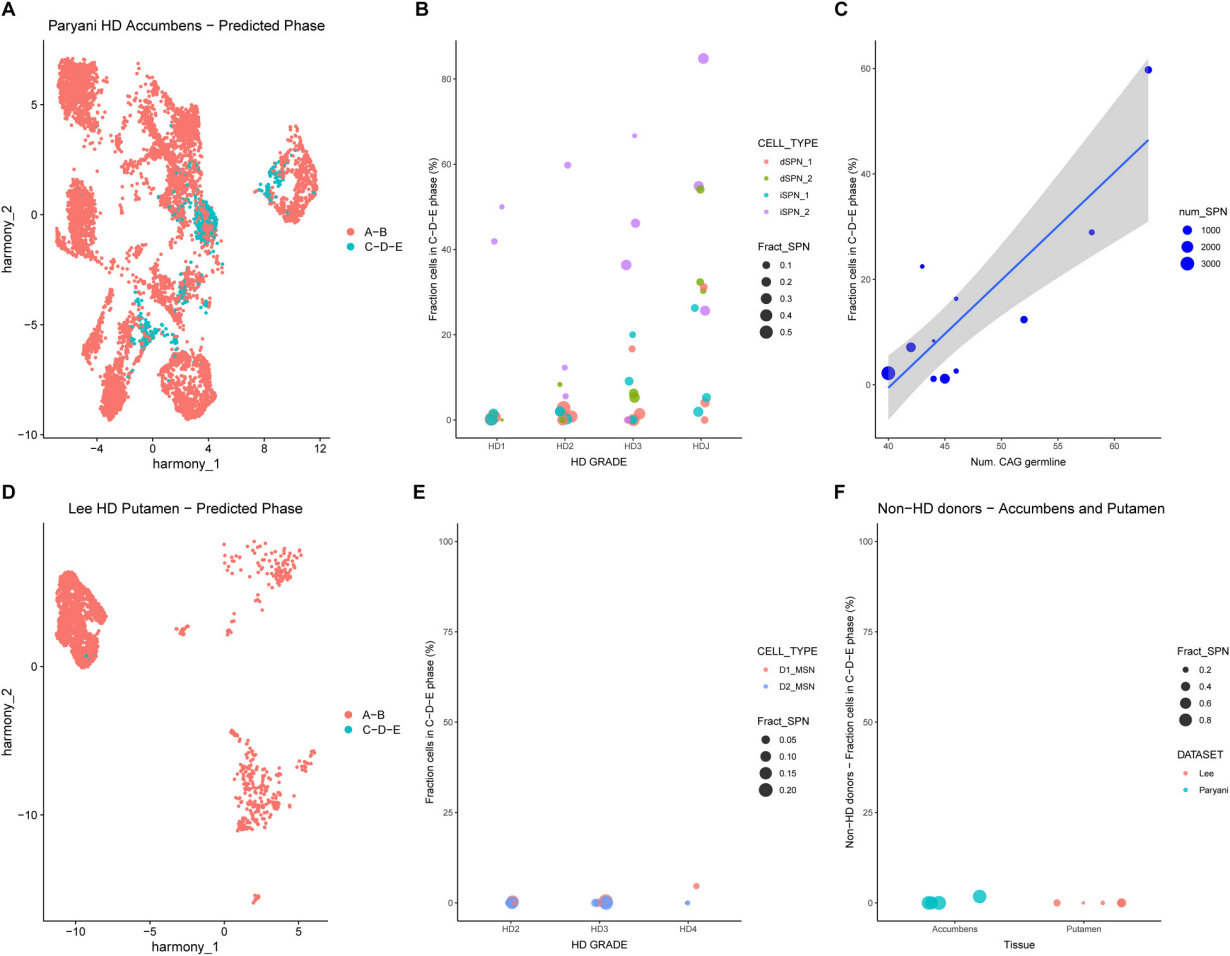
Accumbens and putamen SPNs from external datasets predicted to be in phase ‘C-D-E’ share similar transcriptional profile. (A) UMAP of accumbens SPNs from Paryani et al. coloured by predicted phase. (B) Dotplot showing the fraction of accumbens SPNs from Paryani et al. in ‘C-D-E’ phase for each donor, with the size of each dot proportional to the fraction of SPNs over all cell types. Donors are grouped by HD Grade and are coloured by SPN subtype. (C) Correlation between number of CAG repeats in the germline of each donor and fraction of accumbens SPNs from Paryani et al. in ‘C-D-E’ phase, with the size of each dot proportional to the number of SPNs (r = 0.88, p-value = 0.00, 95% CI: [0.65; 0.97]). (D) UMAP of putamen SPNs from Lee et al. coloured by predicted phase. (E) Dotplot showing the fraction of putamen SPNs from Lee et al. in ‘C-D-E’ phase for each donor, with the size of each dot proportional to the fraction of SPNs over all cell types. Donors are grouped by HD Grade and are coloured by SPN subtype. (F) Dotplot showing the fraction of accumbens and putamen SPNs in ‘C-D-E’ phase for non-HD donors from Paryani et al. and Lee et al., with the size of each dot proportional to the fraction of SPNs over all cell types. Donors are coloured by dataset of origin.

We next asked whether patterns of cell-autonomous transcriptional dysregulation also affect SPNs from other brain regions, such as the accumbens and putamen. The accumbens is located within the ventral striatum and is implicated in reward, motivation, behaviour and movement^30^; while the putamen, together with the caudate, is part of the dorsal striatum and is involved in aspects of goal-directed behaviour.^31^ Although SPNs from such brain regions may have distinct transcriptional profiles compared to caudate SPNs, we challenged our HD-Phase-Model to detect super-expanded SPNs outside the caudate. We subset SPNs from the accumbens of 16 (12 HD, 4 CTRL) and the putamen of 11 (6 HD, 5 CTRL) post-mortem donors described in Paryani et al.^25^ and Lee et al.,^26^ respectively, and visualized their transcriptional profiles via UMAP. As before, we confirmed successful batch integration (Figure S6A,D), examined cell-type distributions based on refined annotations (Figure S6B,E) and annotated cells by Vonsattel grade (Figure S6C,F). We next ran the HD-Phase-Model on these SPNs, subset SPNs from HD donors and coloured cells on the UMAP according to their predicted ELongATE phase (Figure 4A,D). We also plotted UMAPs of accumbens and putamen SPNs stratified by condition (HD vs CTRL), with each SPN coloured according to the inferred probability of being in Phase ‘C-D-E’ (Figure S7A-D), and we reported the distribution of probabilities of SPNs to be in Phase ‘C-D-E’ stratified by condition (Figure S8A-B). As observed in the caudate, only dSPN_2 and iSPN_2 subpopulations from the accumbens of selected donors showed more than 50% of cells predicted to be in Phase ‘C-D-E’ (Figure 4B). Furthermore, the number of *HTT* CAG repeats in the germline of donors from Paryani et al. showed a strong positive correlation with the predicted fraction of Phase ‘C-D-E’ SPNs in accumbens (r = 0.89, p-value = 0.00, 95% CI: [0.65; 0.97]), similarly to the caudate. While, only few SPNs in putamen were predicted to be in Phase ‘C-D-E’, mainly from a single HD4-grade donor (Figure 4E), despite the distribution of probabilities of SPNs to be in Phase ‘C-D-E’ was significantly different between HD and CTRL (Figure S8B) (Wilcoxon test, p-value < 2.2e-16) and the presence of some SPNs in the UMAP of HD donors showing higher probability of being in Phase ‘C-D-E’ compared to CTRLs (Figure S7C-D). Overall, these predictions indicate that SPNs from the accumbens and – to a minor extent – from putamen mimic cell-autonomous transcriptional dysregulation patterns associated with >150 CAG repeats in the *HTT* gene, consistent with what is observed in caudate SPNs. Interestingly, when integrating SPNs from all analysed datasets – covering multiple brain regions – in a single UMAP (Figure S9A) we noticed that predicted super-expanded SPNs still co-localized in the UMAP (Figure S9B), suggesting that cell-autonomous transcriptional dysregulation patterns induced by extreme CAG expansion are shared across multiple brain regions.

### Absence of super-expansion signatures in SPNs from AD and PD donors

**Figure 5.**
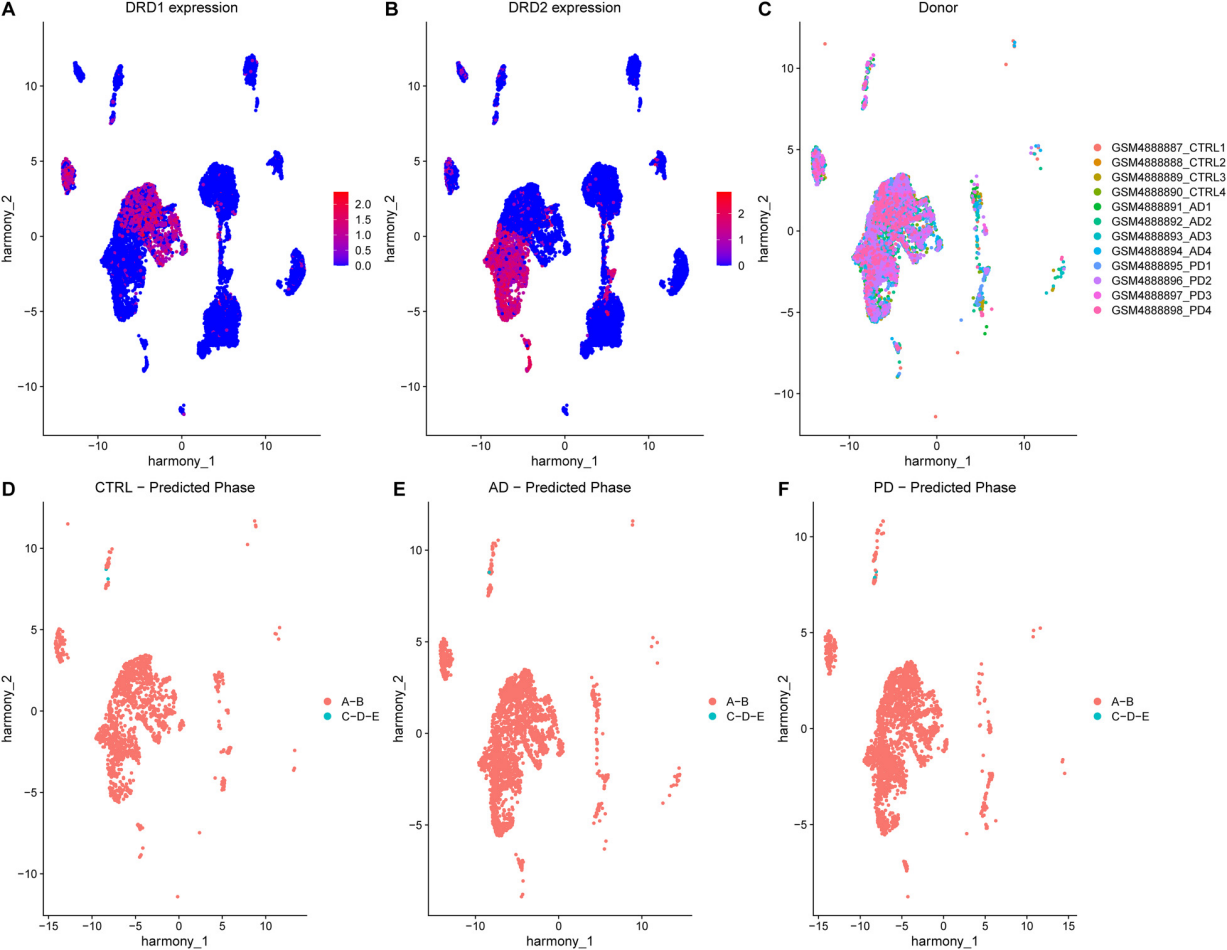
SPNs from AD and PD donors do not show transcriptional dysregulation signatures resembling HD super-expanded SPNs. (A) UMAP of brain cells from Xu et al. coloured by expression levels of DRD1 (legend). (B) UMAP of brain cells from Xu et al. coloured by expression levels of DRD2 (legend). (C) UMAP of SPNs from Xu et al. coloured by donor of origin. (D) UMAP of CTRL SPNs from Xu et al. coloured by predicted phase. (E) UMAP of AD SPNs from Xu et al. coloured by predicted phase. (F) UMAP of PD SPNs from Xu et al. coloured by predicted phase.

After showing that a fraction of SPNs from multiple striatal sub-regions of HD donors are characterized by super-expansion signatures, we asked whether such signatures are also present in SPNs from other neurodegenerative disorders, such as AD and PD, which share some clinical manifestations with HD. We downloaded single-nuclear sequencing data from Xu et al.,^27^ generated from the putamen of post-mortem brains of 4 CTRL, 4 AD and 4 PD donors. After quality control and cell filtering, we visualized the transcriptional profiles of filtered cells using UMAP, colouring cells according to expression levels of the SPN markers DRD1 and DRD2 (Figure 5A,B). Following clustering, we retained only bona-fide SPN clusters and confirmed good integration of cells across all donors (Figure 5C). We applied the HD-Phase-Model to SPNs from all donors (Figure S7E-G, Figure S8C), obtaining 99.87%, 99.96% and 99.82% of SPNs predicted in Phase ‘A-B’ for CTRL, AD and PD donors, respectively (Figure 5D-F). These results support the specificity of the super-expansion signature for HD SPNs, despite shared features of neurodegeneration across HD, AD and PD, and confirm that such signatures do not arise in AD or PD.

## Discussion

More than 30 years after the identification of *HTT* as the gene responsible for HD, compelling evidence from genotyping and sequencing studies points to SI as a central driver of HD pathogenesis.^10^ Despite the availability of multiple bulk and single-nucleus RNA sequencing datasets from HD post-mortem brains and cell lines,^12,13,25,26,32–36^ the presence of super-expanded SPNs in the HD striatum, along with their aberrant transcriptional profile, has remained undetected in traditional case-control studies, likely due to their small fraction within the tissue and the lack of suitable technological and sequencing approaches. This limitation was also evident in the study by Handsaker et al., where a large case-control single-nucleus RNA-seq study including more than 100 donors revealed widespread transcriptional dysregulation in HD brains across all cell types compared to controls, regardless of their propensity to undergo SI.^14^ Remarkably, in an additional dataset generated by the same group - where both *HTT* CAG size and gene expression were measured from the same nuclei - SPNs could be stratified by CAG size, effectively creating a within-sample allelic series. Strikingly, only SPNs with >150 CAG repeats exhibited cell-autonomous transcriptional dysregulation relative to other SPNs from the same donor. These changes followed a consistent, CAG-length-dependent pattern across individuals, with groups of genes showing altered expression levels depending on the *HTT* CAG repeat length within the same cell. In other words, the aberrant transcriptional patterns associated with super-expanded SPNs are layered on top of broader transcriptional alterations observed across the HD brain, likely a consequence of global atrophy, tissue remodeling and devascularization of caudate cells. Importantly, super-expanded SPNs were nearly absent in the brain of two HD donors prior to motor onset, suggesting a potential causal role for these cells in HD neurodegeneration.^14^ These findings support the hypothesis that targeting SI in SPNs may be a promising therapeutic strategy for HD.^16–18^

In our study, we used mathematical modelling to predict the impact of extreme CAG expansions on SPN transcriptional states. *First*, we confirmed that “super-expanded” SPNs in the human post-mortem caudate display consistent transcriptional signatures across donors,^14^ sufficient to drive their co-localization in UMAP space. *Second*, using the only dataset to date with matched transcriptomic and *HTT* CAG sizing data, we trained and validated a two-step model, that we called HD-Phase-Model to identify super-expanded SPNs and estimate their CAG repeat length from gene expression. *Third*, we applied this model to public HD datasets lacking CAG measurements and identified super-expanded SPNs not only in the caudate, but also in accumbens and, to a minor extent, in the putamen. These cells were enriched in HD vs. non-HD donors and consistently clustered together in UMAP space, mirroring results from the training data. Model performance was further supported by the strong correlation between germline *HTT* CAG repeat length and the predicted fraction of super-expanded SPNs at death, consistent with progressive SI accumulation. By contrast, CAP score and Vonsattel HD grade showed non-linear relationships, likely reflecting a dynamic balance: as the fraction of super-expanded SPNs increases, disease severity worsens, yet SPNs that reach the “Elimination” phase of the ELongATE model are more likely to die, reducing their measurable fraction at later stages.

*Fourth*, we assessed whether transcriptional dysregulations in SPNs from AD and PD donors resembled that of HD super-expanded SPNs. Indeed, we found that virtually no SPNs from AD or PD shared these transcriptional profiles, reinforcing the specificity of the HD signature. The model also offered a molecular explanation for previously uncharacterized subclusters, such as dSPN_2 and iSPN_2.^25^ As single-nuclei datasets for SPNs from related CAG·CTG expansion disorders become available, the model will also provide insights into shared and gene-specific patterns associated with super-expansions.

Together, our findings show that AI-based models can accurately predict *HTT* CAG repeat length from single-nucleus transcriptomics, providing a powerful tool to dissect transcriptional heterogeneity in HD single cell datasets lacking CAG information. While our proof-of-concept study did not benchmark multiple algorithms and relied solely on logistic and linear regression, we believe that the adoption of more advanced AI methods could further improve classification performances. Although trained on caudate SPNs, our model generalized quite well to other brain regions, including accumbens and putamen. Nevertheless, developing region-specific models trained on matched data from these areas will further enhance predictive power and resolution. The need for region-specific models is further supported by a recent preprint from McCarroll's lab, which describes cell-autonomous transcriptional dysregulation above the 150 CAG repeats threshold in cortical neurons, albeit involving a different set of Phase C and Phase D genes compared to striatal neurons.^37^

Ultimately, generating additional datasets combining transcriptomic profiling and CAG sizing from the same SPNs – while controlling for technical variability – will be crucial to build robust predictive CAG sizing models. Such tools will refine our understanding of HD pathogenesis and guide the design of future therapeutic strategies targeting somatic instability.

## Supplemental Material

sj-pdf-1-hun-10.1177_18796397261443137 - Supplemental material for Towards AI-driven prediction of *HTT* CAG size in super-expanded human spiny projection neurons from Huntington disease donors Supplemental material, sj-pdf-1-hun-10.1177_18796397261443137 for Towards AI-driven prediction of *HTT* CAG size in super-expanded human spiny projection neurons from Huntington disease donors by Simone Maestri, Davide Scalzo, Martina Zobel, Dario Besusso and Elena Cattaneo in Journal of Huntington's Disease

sj-pdf-2-hun-10.1177_18796397261443137 - Supplemental material for Towards AI-driven prediction of *HTT* CAG size in super-expanded human spiny projection neurons from Huntington disease donors Supplemental material, sj-pdf-2-hun-10.1177_18796397261443137 for Towards AI-driven prediction of *HTT* CAG size in super-expanded human spiny projection neurons from Huntington disease donors by Simone Maestri, Davide Scalzo, Martina Zobel, Dario Besusso and Elena Cattaneo in Journal of Huntington's Disease

sj-xlsx-3-hun-10.1177_18796397261443137 - Supplemental material for Towards AI-driven prediction of *HTT* CAG size in super-expanded human spiny projection neurons from Huntington disease donors Supplemental material, sj-xlsx-3-hun-10.1177_18796397261443137 for Towards AI-driven prediction of *HTT* CAG size in super-expanded human spiny projection neurons from Huntington disease donors by Simone Maestri, Davide Scalzo, Martina Zobel, Dario Besusso and Elena Cattaneo in Journal of Huntington's Disease
