# Supplementary material for: Towards AI-driven prediction of HTT CAG size in super-expanded human spiny projection neurons from Huntington disease donors
Source: J Huntingtons Dis. 2026 Apr 20;15(3):408–20. doi: 10.1177/18796397261443137 (PMC13478745; doi:10.1177/18796397261443137)
Supplement: Supplementary file 1 [file sj-pdf-1-hun-10.1177_18796397261443137.pdf]

TOWARDS AI-DRIVEN PREDICTION OF *HTT* CAG SIZE IN SUPER-EXPANDED HUMAN SPINY PROJECTION NEURONS FROM HUNTINGTON DISEASE DONORS

Simone Maestri*[1,2], Davide Scalzo[1,2], Martina Zobel[1,2], Dario Besusso[1,2], Elena Cattaneo[1,2]

1. Dipartimento di Bioscienze, Università degli Studi di Milano, 20133 Milano
2. Istituto Nazionale di Genetica Molecolare

*Corresponding author: simone.maestri@unimi.it

SUPPLEMENTARY FIGURES CAPTIONS

Figure S1. QC and preprocessing of caudate SPNs from Handsaker et al. (A) UMAP of SPNs coloured by donor. (B) UMAP of SPNs coloured by number of expressed genes. (C) UMAP of SPNs coloured by number of detected molecules. (D) UMAP of SPNs coloured by percentage mitochondrial genes. (E) UMAP of SPNs coloured by CAG size. (F) UMAP of SPNs coloured by cluster.

Figure S2. HD-Phase-Model training and prediction. (A) Training procedure for Phase model using single-nuclei data for SPNs from Handsaker et al. (B) Training procedure for CAG sizing model using single-nuclei data for SPNs from Handsaker et al. (C) Procedure for predicting phase and CAG size from single-nuclei SPN datasets, and for checking their accuracy, in case CAG size measures are available.

Figure S3. QC and preprocessing of caudate SPNs from Paryani et al. and from Lee et al. (A) UMAP of caudate SPNs from Paryani et al. coloured by Batch. (B) UMAP of caudate SPNs from Paryani et al. coloured by SPN subtype. (C) UMAP of caudate SPNs from Paryani et al. coloured by HD Grade. (D) UMAP of caudate SPNs from Lee et al. coloured by Batch. (E) UMAP of caudate SPNs from Lee et al. coloured by SPN subtype. (E) UMAP of caudate SPNs from Lee et al. coloured by HD Grade.

Figure S4. QC and preprocessing of non-HD SPNs from Paryani et al. and from Lee et al. (A) UMAP of non-HD caudate SPNs from Handsaker et al. coloured by predicted phase. (B) UMAP of non-HD caudate SPNs from Paryani et al. coloured by predicted phase. (C) UMAP of non-HD caudate SPNs from Lee et al. coloured by predicted phase. (D) UMAP of non-HD accumbens SPNs from Paryani et al. coloured by predicted phase. (E) UMAP of non-HD putamen SPNs from Lee et al. coloured by predicted phase. (F) Dotplot showing the fraction of SPNs in ‘C-D-E’ phase for non-HD donors from Handsaker et al, Paryani et al. and Lee et al., with the size of each dot proportional to the fraction of SPNs over all cell types. Donors are coloured by dataset of origin and grouped by tissue.

Figure S5. Predicted classification probability of Phase ‘C-D-E’ for caudate SPNs stratified by condition. (A) UMAP of caudate HD SPNs from Handsaker et al. (B) UMAP of caudate CTRL SPNs from Handsaker et al. (C) UMAP of caudate HD SPNs from Paryani et al. (D) UMAP of caudate CTRL SPNs from Paryani et al. (E) UMAP of caudate HD SPNs from Lee et al. (F) UMAP of caudate CTRL SPNs from Lee et al.

Figure S6. QC and preprocessing of accumbens and putamen SPNs from Paryani et al. and from Lee et al. (A) UMAP of accumbens SPNs from Paryani et al. coloured by Batch. (B) UMAP of accumbens SPNs from Paryani et al.

coloured by SPN subtype. (C) UMAP of accumbens SPNs from Paryani et al. coloured by HD Grade. (D) UMAP of putamen SPNs from Lee et al. coloured by Batch. (E) UMAP of putamen SPNs from Lee et al. coloured by SPN subtype. (E) UMAP of putamen SPNs from Lee et al. coloured by HD Grade.

Figure S7. Predicted classification probability of Phase 'C-D-E' for accumbens and putamen SPNs stratified by condition. (A) UMAP of accumbens HD SPNs from Paryani et al. (B) UMAP of accumbens CTRL SPNs from Paryani et al. (C) UMAP of putamen HD SPNs from Lee et al. (D) UMAP of putamen CTRL SPNs from Lee et al. (E) UMAP of putamen CTRL SPNs from Xu et al. (F) UMAP of putamen AD SPNs from Xu et al. (G) UMAP of putamen PD SPNs from Xu et al.

Figure S8. Distribution of predicted classification probability of Phase 'C-D-E' for accumbens and putamen SPNs stratified by condition. (A) Violin plot showing the distribution of classification probabilities of accumbens HD and CTRL SPNs from Paryani et al. (Wilcoxon test, HD vs Control, p-value < 2.2e-16) (B) Violin plot showing the distribution of classification probabilities of putamen HD and CTRL SPNs from Lee et al. (Wilcoxon test, HD vs Control, p-value < 2.2e-16). (C) Violin plot showing the distribution of classification probabilities of putamen AD, PD and CTRL SPNs from Xu et al (Wilcoxon test, AD vs Control, p-value < 2.2e-16; PD vs Control, p-value = 0.1133).

Figure S9. Transcriptional profile of all post-mortem HD donors across datasets and brain regions. (A) UMAP of HD donors with cells colored by dataset of origin. (B) UMAP of HD donors with cells colored by predicted phase of the ELongATE model.
