# Supplementary material for: Towards AI-driven prediction of HTT CAG size in super-expanded human spiny projection neurons from Huntington disease donors
Source: J Huntingtons Dis. 2026 Apr 20;15(3):408–20. doi: 10.1177/18796397261443137 (PMC13478745; doi:10.1177/18796397261443137)
Supplement: Supplementary file 2 [file sj-pdf-2-hun-10.1177_18796397261443137.pdf]

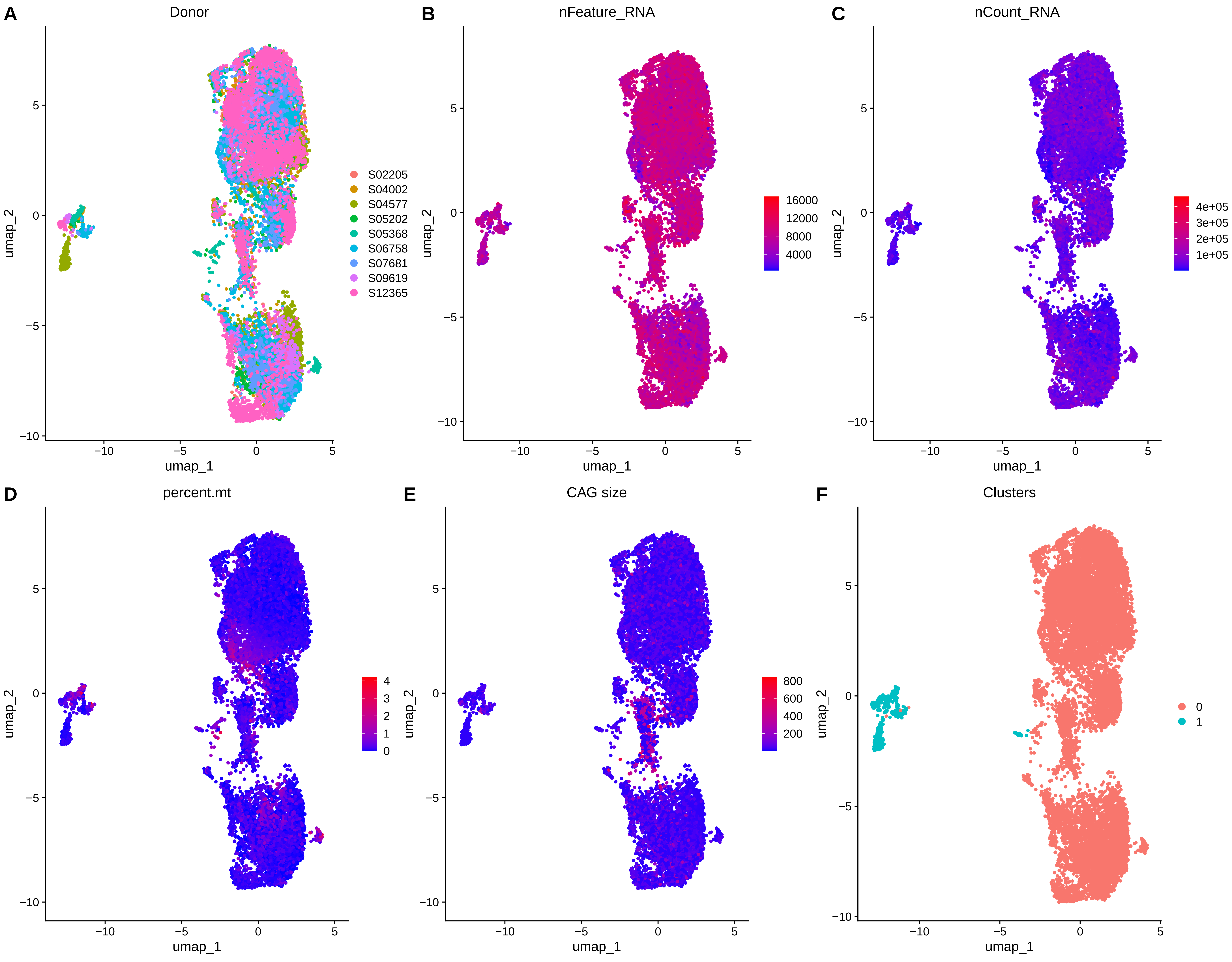

A
Donor
S02205
S04002
S04577
S05202
S05368
S06758
S07681
S09619
S12365
umap_1
umap_2
B
nFeature_RNA
16000
12000
8000
4000
C
nCount_RNA
4e+05
3e+05
2e+05
1e+05
D
percent.mt
4
3
2
1
0
E
CAG size
800
600
400
200
F
Clusters
0
1

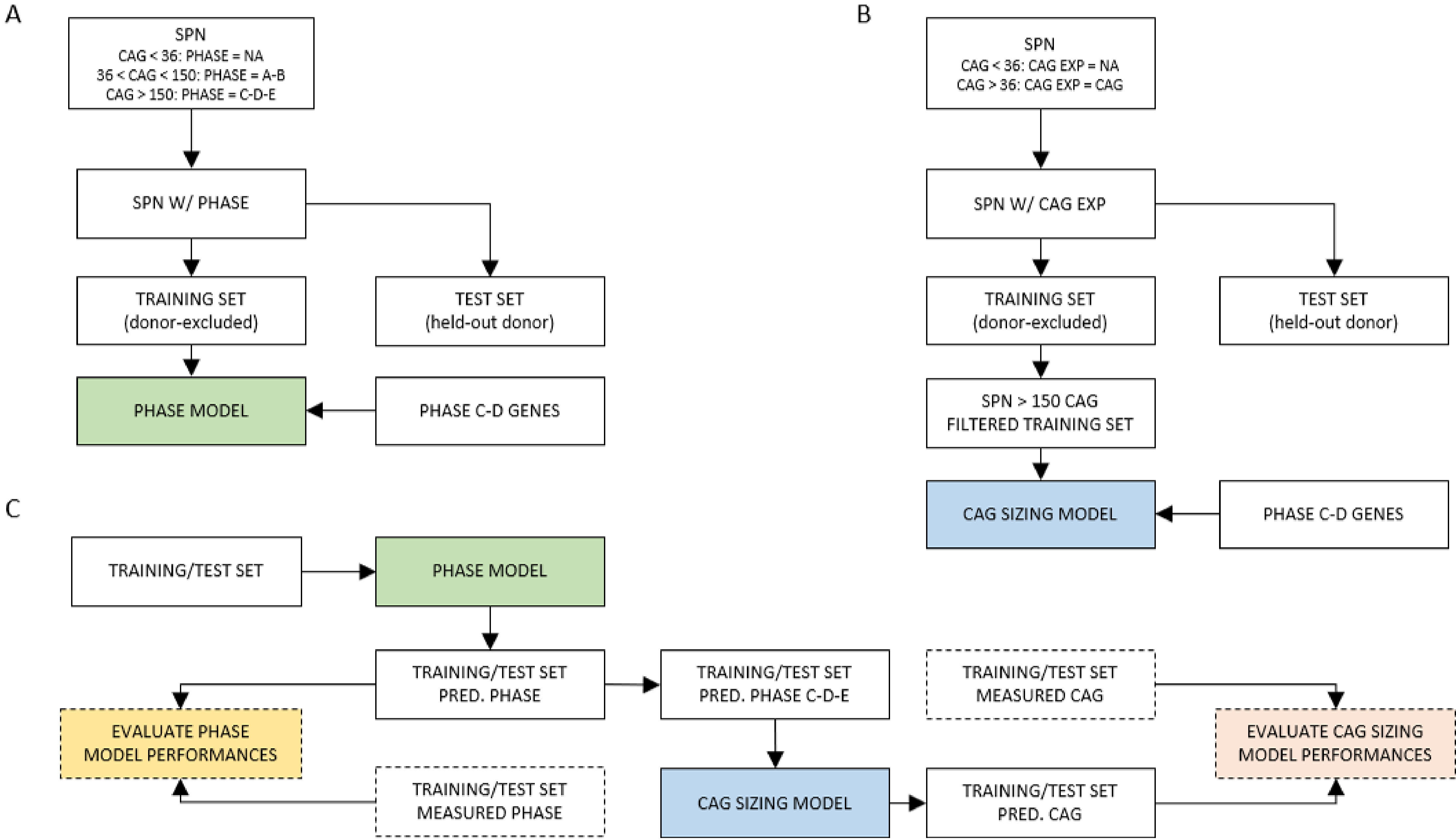
A
SPN
CAG < 36: PHASE = NA
36 < CAG < 150: PHASE = A-B
CAG > 150: PHASE = C-D-E
SPN W/ PHASE
TRAINING SET
(donor-excluded)
TEST SET
(held-out donor)
PHASE MODEL
PHASE C-D GENES
B
SPN
CAG < 36: CAG EXP = NA
CAG > 36: CAG EXP = CAG
SPN W/ CAG EXP
TRAINING SET
(donor-excluded)
TEST SET
(held-out donor)
SPN > 150 CAG
FILTERED TRAINING SET
CAG SIZING MODEL
PHASE C-D GENES
C
TRAINING/TEST SET
PHASE MODEL
TRAINING/TEST SET
PRED. PHASE
TRAINING/TEST SET
PRED. PHASE C-D-E
TRAINING/TEST SET
MEASURED CAG
EVALUATE PHASE
MODEL PERFORMANCES
EVALUATE CAG SIZING
MODEL PERFORMANCES
TRAINING/TEST SET
MEASURED PHASE
CAG SIZING MODEL
TRAINING/TEST SET
PRED. CAG

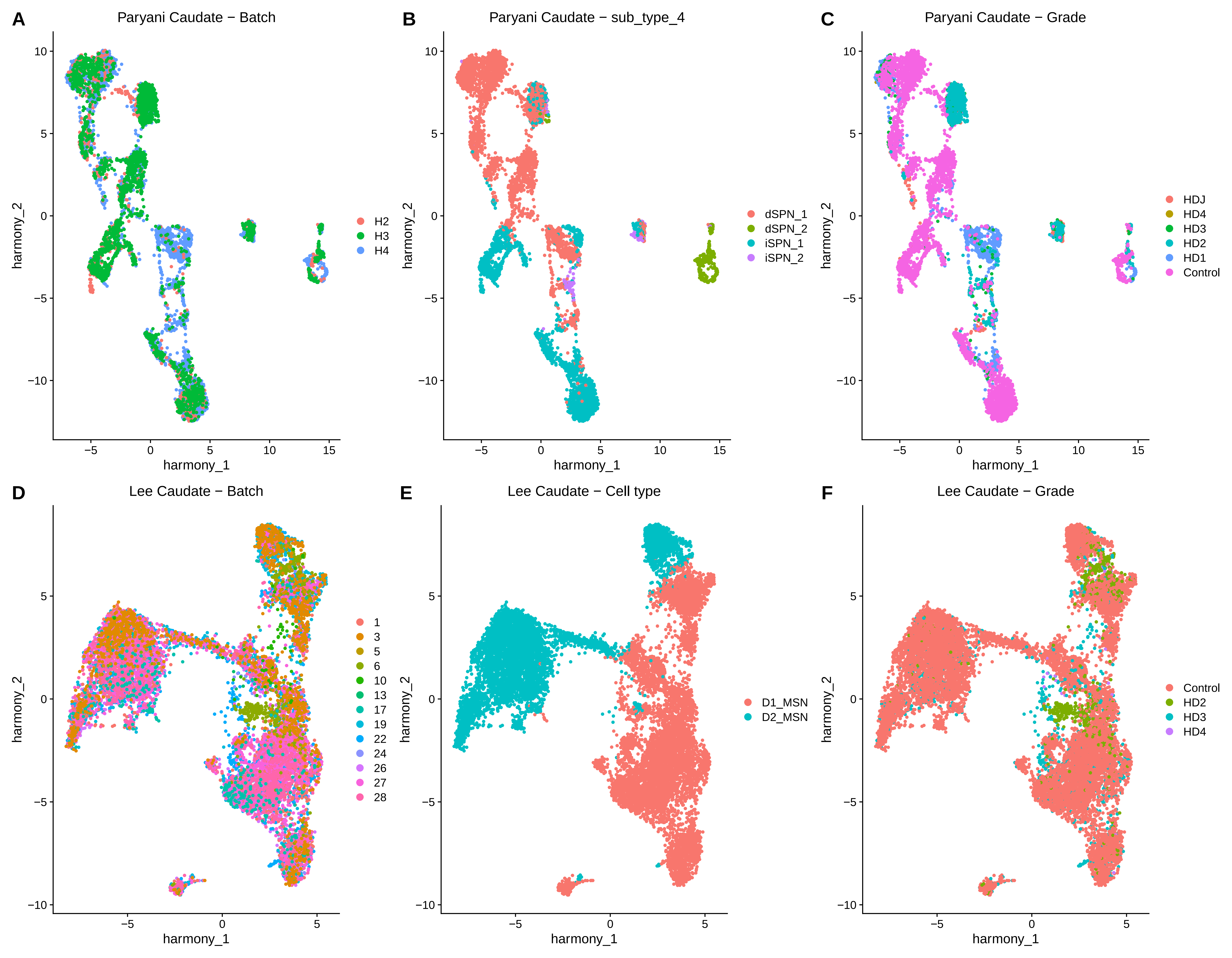

A
Paryani Caudate − Batch
H2
H3
H4
B
Paryani Caudate − sub_type_4
dSPN_1
dSPN_2
iSPN_1
iSPN_2
C
Paryani Caudate − Grade
HDJ
HD4
HD3
HD2
HD1
Control
D
Lee Caudate − Batch
1
3
5
6
10
13
17
19
22
24
26
27
28
E
Lee Caudate − Cell type
D1_MSN
D2_MSN
F
Lee Caudate − Grade
Control
HD2
HD3
HD4
harmony_1
harmony_2

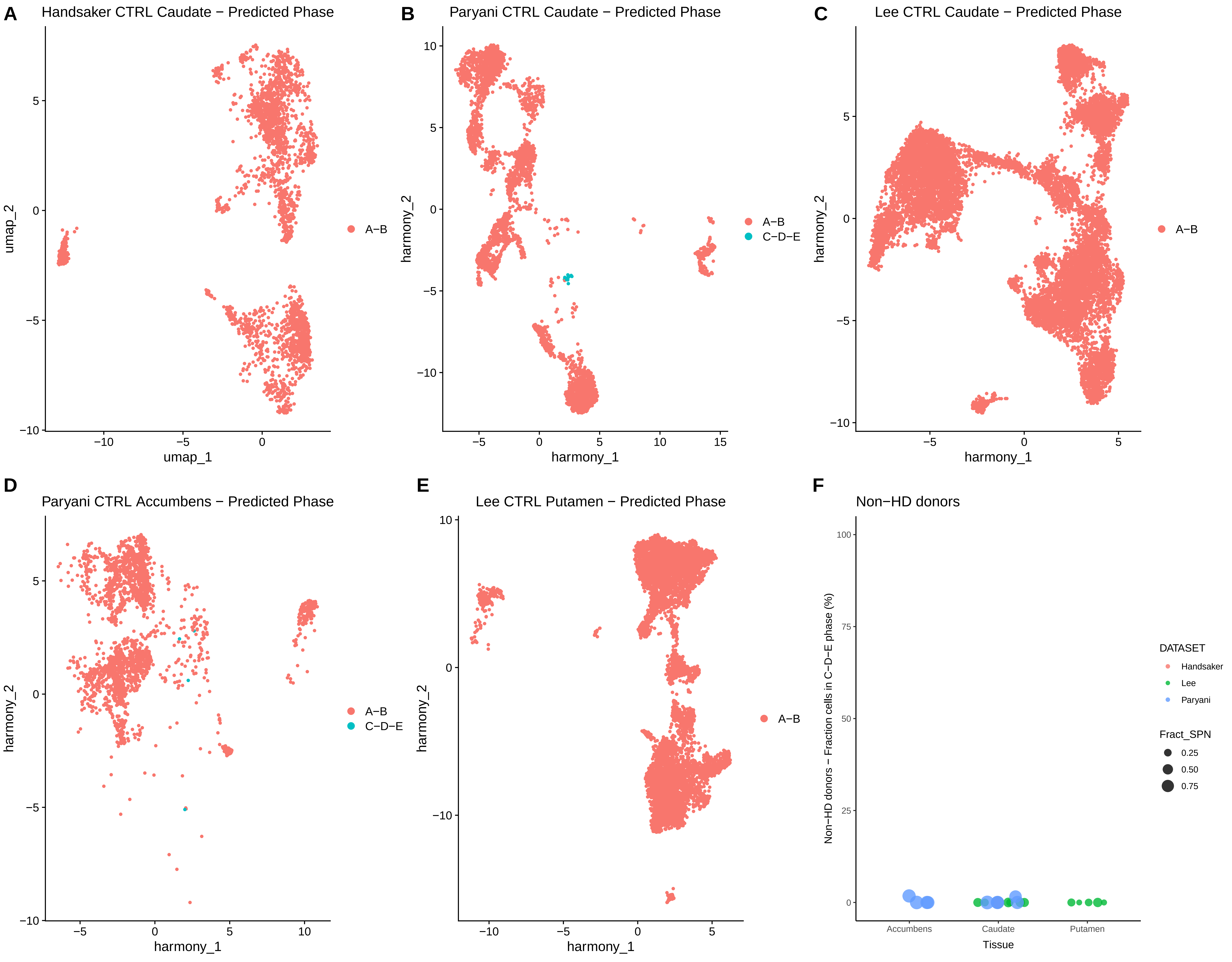

A
Handsaker CTRL Caudate − Predicted Phase
umap_1
umap_2
A−B
B
Paryani CTRL Caudate − Predicted Phase
harmony_1
harmony_2
A−B
C−D−E
C
Lee CTRL Caudate − Predicted Phase
harmony_1
harmony_2
A−B
D
Paryani CTRL Accumbens − Predicted Phase
harmony_1
harmony_2
A−B
C−D−E
E
Lee CTRL Putamen − Predicted Phase
harmony_1
harmony_2
A−B
F
Non−HD donors
Non−HD donors − Fraction cells in C−D−E phase (%)
Accumbens
Caudate
Putamen
Tissue
DATASET
Handsaker
Lee
Paryani
Fract_SPN
0.25
0.50
0.75

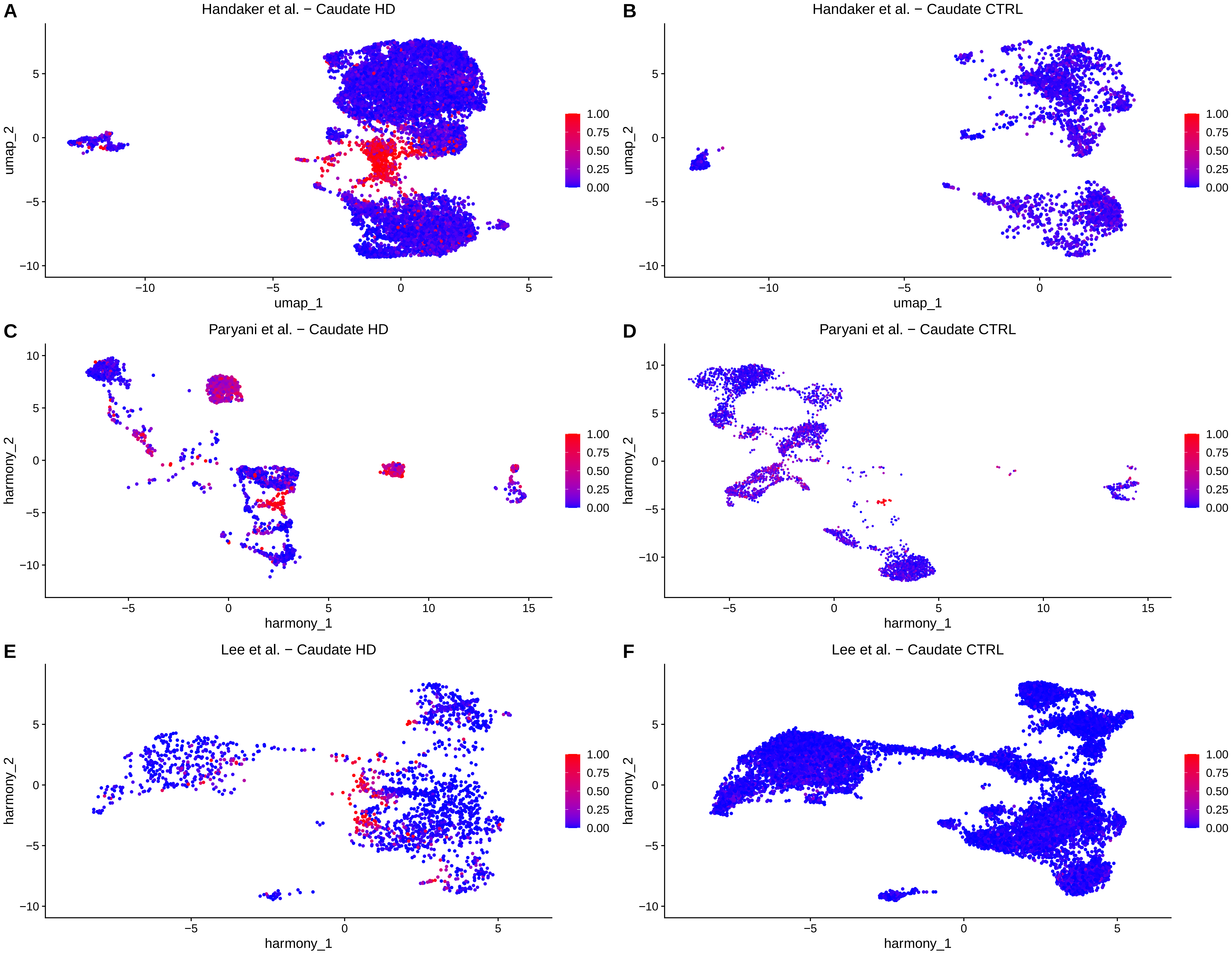
A
Handaker et al. − Caudate HD
B
Handaker et al. − Caudate CTRL
C
Paryani et al. − Caudate HD
D
Paryani et al. − Caudate CTRL
E
Lee et al. − Caudate HD
F
Lee et al. − Caudate CTRL
umap_1
umap_2
harmony_1
harmony_2
1.00
0.75
0.50
0.25
0.00

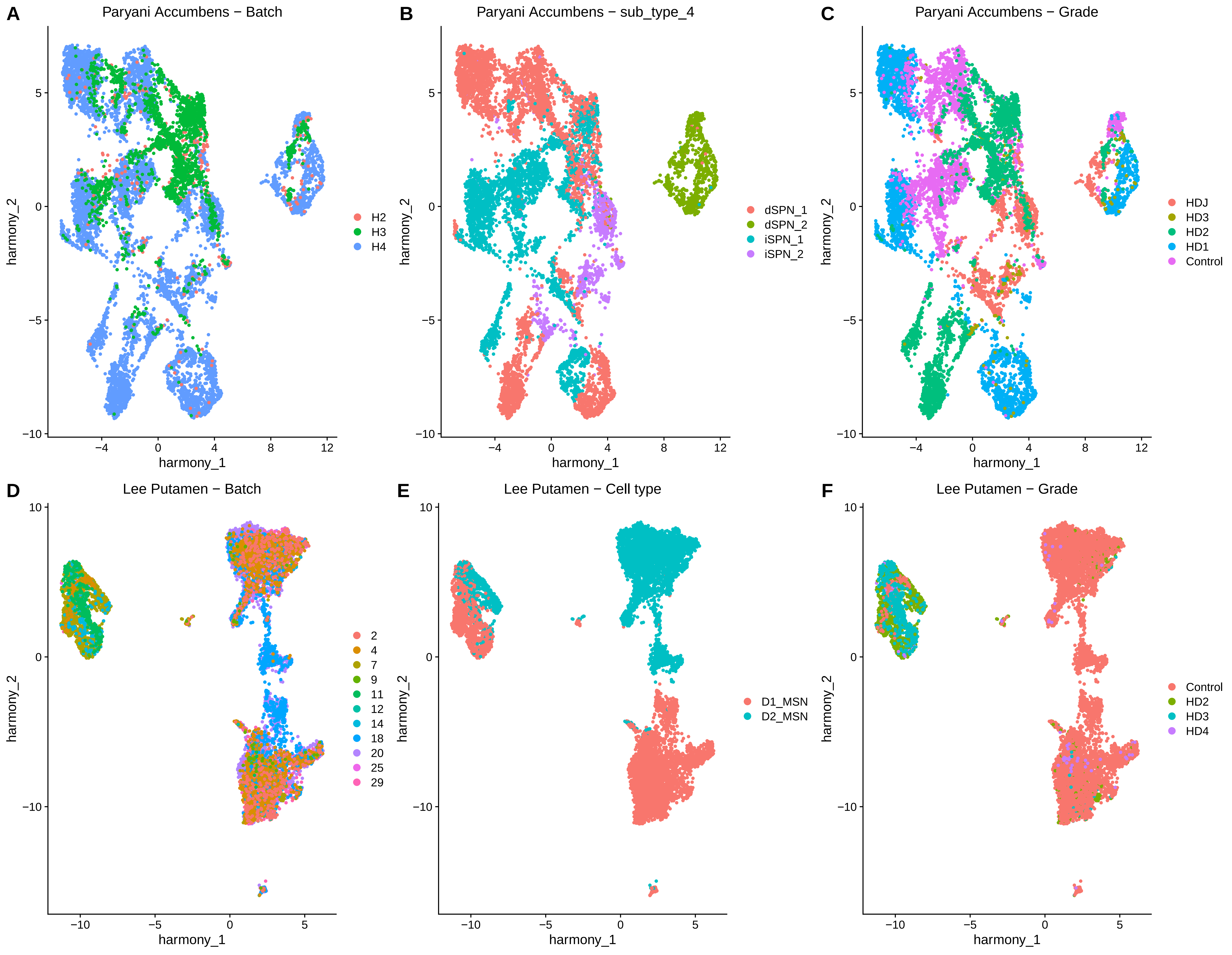

A
Paryani Accumbens − Batch
H2
H3
H4
B
Paryani Accumbens − sub_type_4
dSPN_1
dSPN_2
iSPN_1
iSPN_2
C
Paryani Accumbens − Grade
HDJ
HD3
HD2
HD1
Control
D
Lee Putamen − Batch
2
4
7
9
11
12
14
18
20
25
29
E
Lee Putamen − Cell type
D1_MSN
D2_MSN
F
Lee Putamen − Grade
Control
HD2
HD3
HD4
harmony_1
harmony_2

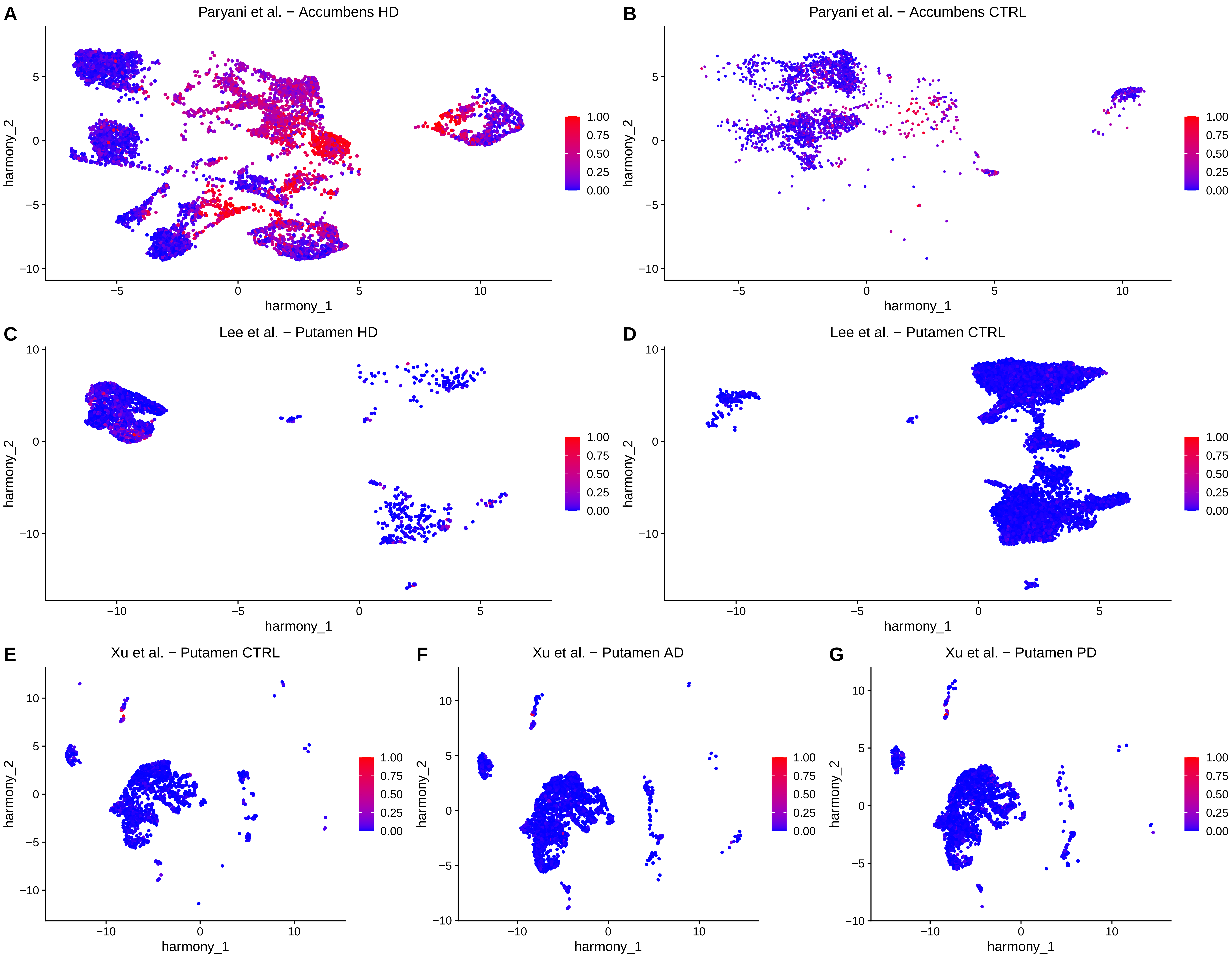
A
Paryani et al. − Accumbens HD
B
Paryani et al. − Accumbens CTRL
C
Lee et al. − Putamen HD
D
Lee et al. − Putamen CTRL
E
Xu et al. − Putamen CTRL
F
Xu et al. − Putamen AD
G
Xu et al. − Putamen PD
harmony_1
harmony_2
1.00
0.75
0.50
0.25
0.00

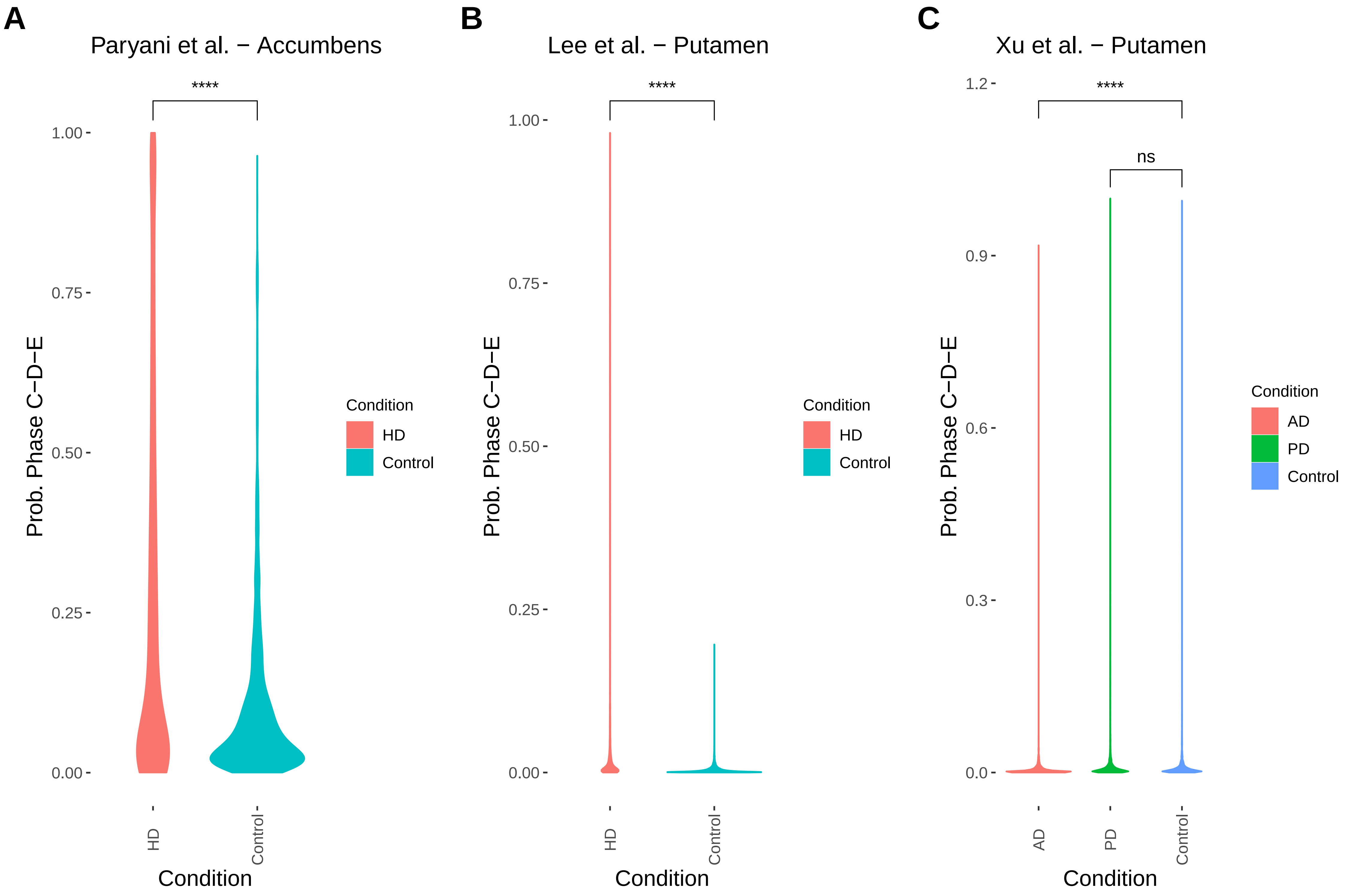

A
Paryani et al. − Accumbens
****
1.00
0.75
0.50
0.25
0.00
Prob. Phase C−D−E
HD
Control
Condition
Condition
HD
Control
B
Lee et al. − Putamen
****
1.00
0.75
0.50
0.25
0.00
Prob. Phase C−D−E
HD
Control
Condition
Condition
HD
Control
C
Xu et al. − Putamen
****
ns
1.2
0.9
0.6
0.3
0.0
Prob. Phase C−D−E
AD
PD
Control
Condition
Condition
AD
PD
Control

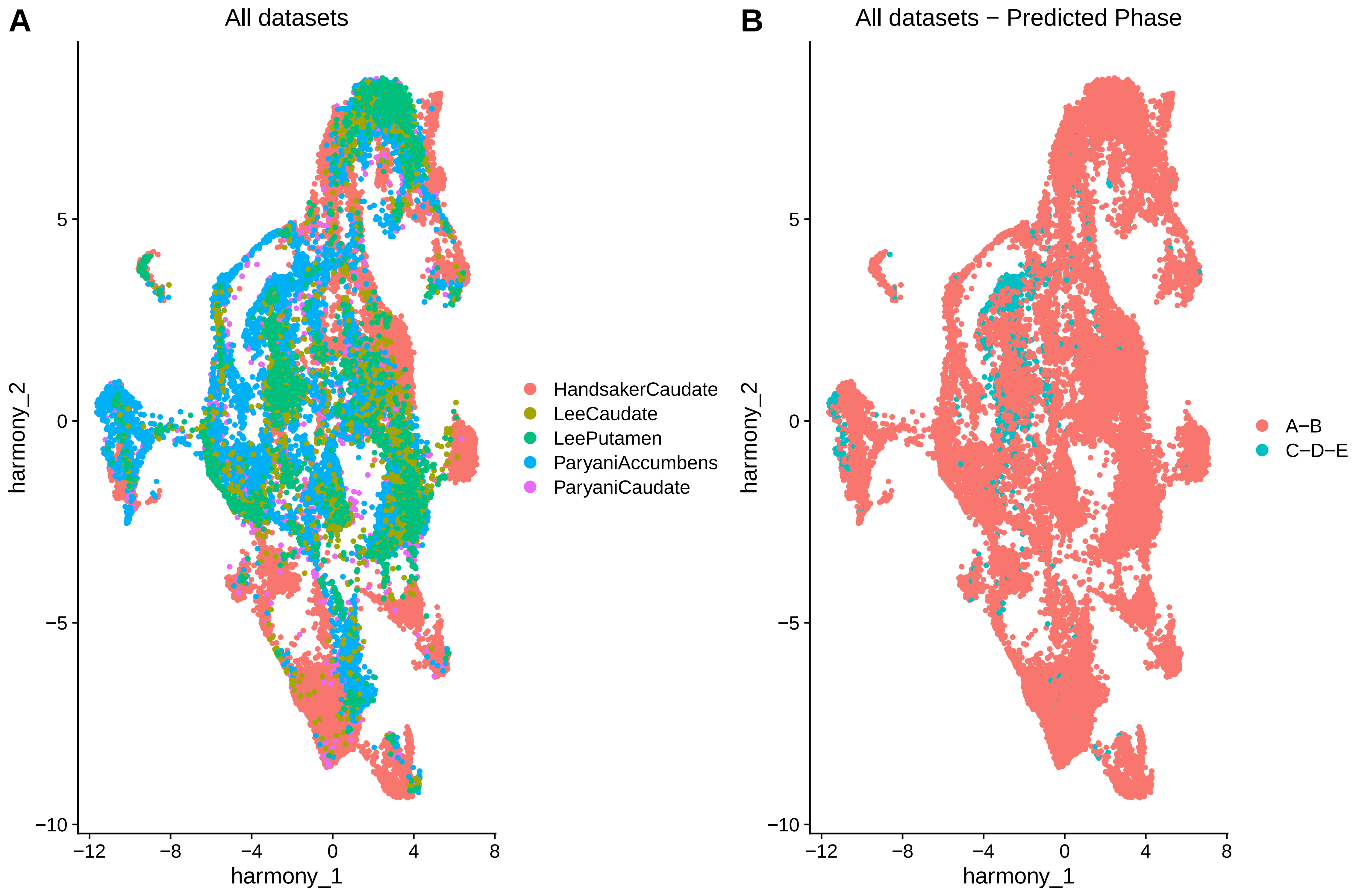

A
All datasets
B
All datasets − Predicted Phase
harmony_2
harmony_1
5
0
−5
−10
−12
−8
−4
0
4
8
HandsakerCaudate
LeeCaudate
LeePutamen
ParyaniAccumbens
ParyaniCaudate
A−B
C−D−E
